# Checklist- and algorithm-based simulation training produce comparable improvements in teamwork-related shared mental models: a cluster-randomized study in trauma resuscitation teams

**DOI:** 10.1007/s00068-026-03161-0

**Published:** 2026-04-02

**Authors:** Yvonne Beaugé, Simon Kusch, Lukas Thorsson, Michael Zentgraf, Julian Schauwienold, Matthias Muenzberg, Rene Verboket, Rolf Lefering, Uwe Schweigkofler, Philipp Faul, Jasmina Sterz, Miriam Ruesseler

**Affiliations:** 1 Goethe University Frankfurt, Medical Faculty, Institute for Medical Education and Clinical Simulation, Theodor-Stern Kai 7, 60590 Frankfurt/Main, Germany; 2 Berufsgenossenschaftliche Unfallklinik, 60389 Frankfurt/Main, Germany; 3 Goethe University Frankfurt, University Hospital, Department for Trauma and Orthopedic Surgery, Theodor Stern Kai 7, 60590 Frankfurt/Main, Germany; 4 Institute for Research in Operative Medicine, Faculty of Health, Ostmerheimer Str. 200, 51109 Köln, Germany

**Keywords:** Shared mental models, Trauma resuscitation, Simulation training, Interprofessional teamwork, Checklists, Algorithm-based training

## Abstract

**Purpose:**

Shared mental models (SMMs) reflect the extent to which team members hold a similar understanding of roles and team processes. In trauma resuscitation, higher SMM alignment is considered a prerequisite for coordinated interprofessional teamwork. This study compared the effects of checklist-based versus algorithm-based in situ simulation training on teamwork-related SMMs in trauma room teams.

**Methods:**

In this multicenter, cluster-randomized pre–post study, 29 interprofessional trauma teams (186 participants) from six hospitals received either checklist-based training (*n* = 15 teams) incorporating the Trauma Room Manual checklists or algorithm-based training (*n* = 14 teams), both grounded in ATLS principles. Teams completed an SMM questionnaire adapted to the trauma setting, based on a previously published instrument, assessing similarity in (1) task responsibility and (2) team communication. Team-level similarity scores were calculated pre- and post-training. Training effects were analyzed using mixed repeated-measures ANOVA.

**Results:**

Across both training formats, teamwork-related shared mental models increased significantly from pre- to post-training in total similarity, task responsibility, and communication. No significant between-group differences or time × group interactions were observed. The interaction for task responsibility approached significance (*p* =.06; η²*p* =.12).

**Conclusion:**

A single-day in situ simulation training improved teamwork-related shared mental models in interprofessional trauma teams. Checklist- and algorithm-based formats produced similar short-term gains. Future studies should evaluate long-term retention, implementation fidelity, and whether improved SMM alignment translates into measurable team performance and patient-safety outcomes.

## Introduction

Effective trauma care requires rapid decision-making, coordinated team performance, and high reliability under extreme time pressure [1, 2]. The management of polytrauma patients depends on interdisciplinary teams operating in environments characterized by complexity, unpredictability, and urgency. In the German healthcare system, trauma teams typically comprise at least two physicians and two specialized nursing staff [3], who must master both technical and non-technical skills, including structured communication, situational awareness, and adaptive teamwork [4, 5].

Human factors and teamwork failures remain important contributors to error and inefficiency in trauma resuscitation, despite continued advances in diagnostics, protocols, and organizational standards [6]. Shared mental models (SMMs), a shared understanding of roles, responsibilities, and team interaction patterns, are considered a key cognitive mechanism supporting coordinated action in teams [7, 8]. Misalignment in such shared representations can plausibly contribute to communication breakdowns, duplicated or omitted tasks, and delays in critical interventions. In trauma settings, these phenomena are often captured through process metrics and structured performance analyses, including video review [9, 10].

Structured approaches to improve trauma team performance include Standard Operating Procedures, team training, and simulation-based programs [6]. The Advanced Trauma Life Support (ATLS^®^) framework provides a widely adopted algorithm-based structure (ABCDE), role definition, and task prioritization intended to support shared situational awareness and coordination [2, 5]. However, algorithmic guidance alone does not necessarily ensure explicit, team-wide verification of critical steps. Checklists add structured verbalization and cross-checking (e.g., read–do, challenge–response), aiming to reduce workflow deviations and task omissions in high-risk perioperative and critical care settings [11–14]. In trauma resuscitation specifically, checklist use has been associated with fewer deviations from ATLS workflows [15]. The Trauma Room Manual (TRM) integrates such checklists to standardize trauma room workflows and communication checkpoints [16].

Although checklists are increasingly implemented in trauma systems, their incremental contribution to team cognition, specifically, to teamwork-related SMM alignment, remains insufficiently understood. Empirical work in healthcare teams suggests that structured team training and standardization efforts (including simulation-based interventions) can promote shared cognitive alignment and coordination, although designs and measures vary substantially [17–19]. Conceptual and theoretical work further supports the relevance of shared mental models and cognitive aids for coordination in high-risk environments [20, 21]. In trauma teams, related constructs such as transactive memory systems have also been linked to performance under time pressure, highlighting the broader importance of shared cognitive structures for effective action teams [22]. Yet it remains unclear whether adding checklist-based cognitive aids to otherwise standardized algorithm-based training yields incremental gains in teamwork-related SMM alignment.

This cluster-randomized investigation compares checklist-based and algorithm-based in situ simulation training to determine their effects on teamwork-related shared mental models in interprofessional trauma teams. Specifically, it evaluated whether structured simulation training increased SMM alignment over time and whether integrating checklist-based cognitive aids produced greater improvements in task responsibility and team communication. By isolating instructional format within an otherwise standardized framework, the analysis clarifies the contribution of checklist-based cognitive support to shared team cognition in trauma resuscitation.

## Methods

### Study design

**Fig. 1:**
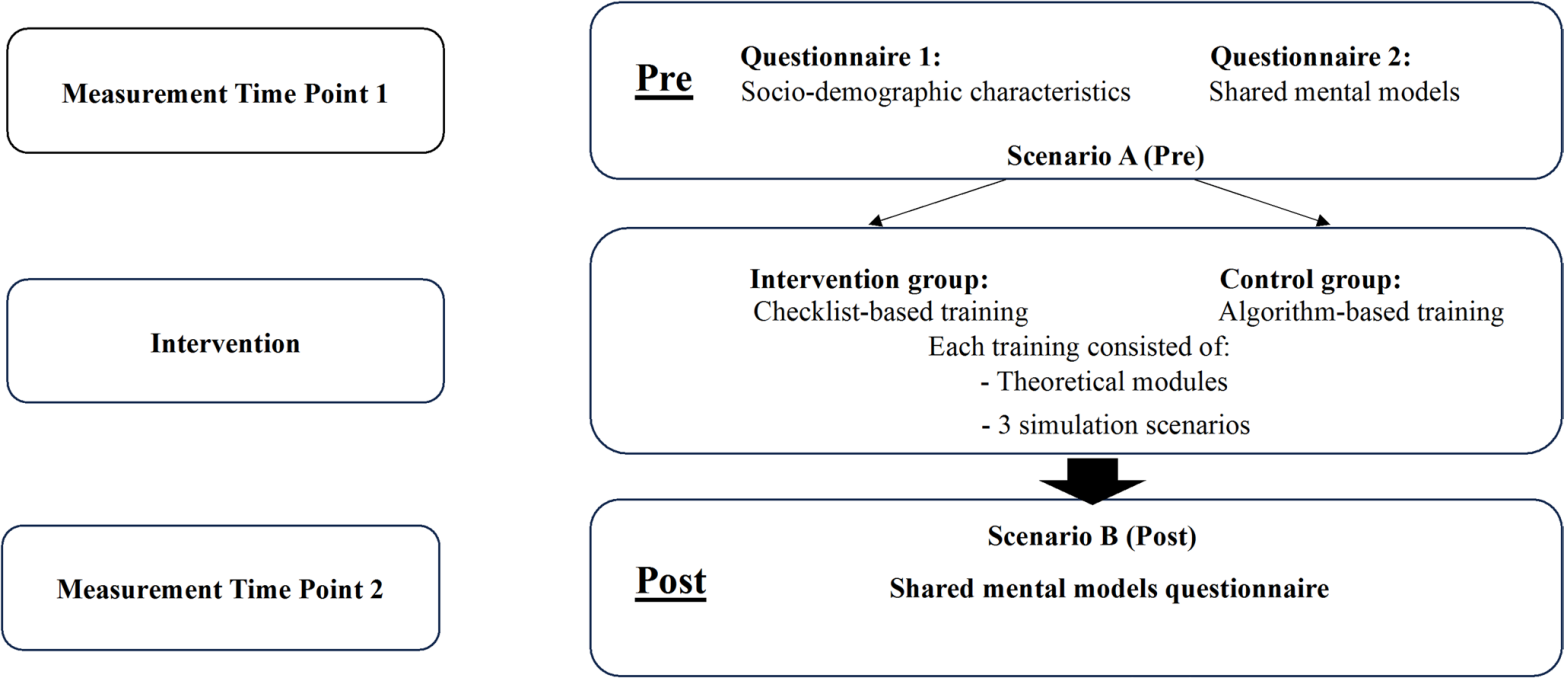
Study design

This cluster-randomized multicenter study employed a pre-post design to compare the effectiveness of checklist-based versus algorithm-based training in enhancing SMM among trauma room teams **(**Fig. 1**).** Teams were randomized using coin-flip allocation immediately prior to the intervention. After allocation, each trauma team received the assigned training intervention.

Blinding to group allocation was not feasible due to the nature of the intervention. To minimize potential bias, participants were not informed about the study objectives or the two-arm design.

Written informed consent was obtained from all participants prior to enrollment, who were free to withdraw at any time. All data were anonymized. According to the Ethics Committee of the Faculty of Medicine at Goethe University, this study did not require formal ethical approval and was conducted in accordance with the principles outlined in the Declaration of Helsinki.

### Study participants

Participants were healthcare professionals involved in polytrauma care from participating hospitals, including emergency and anesthesia nurses, medical-technical radiology assistants, and physicians from trauma surgery, anesthesiology, radiology, and visceral surgery. Recruitment was initiated via the TraumaNetwork DGU^®^ Südhessen, Germany, and supplemented by direct contact with hospital leadership and professional networks.

Inclusion criteria required a minimum of six months of trauma room experience and consent to audio and video recording during simulation training. Teams were excluded if mandatory team members were missing at the time of the simulation.

### Sample size

An a priori sample size calculation was performed using G*Power for a within-subjects pre–post comparison, assuming a medium effect size (d = 0.5), α = 0.05, and power = 0.80, which indicated that 33 teams would be required. This calculation was intended to provide an approximate recruitment target in the absence of prior effect size estimates for checklist-related changes in trauma team SMM. Because outcomes were analyzed at the team level and the study followed an exploratory cluster-randomized design, this calculation was used for planning purposes rather than as a strict power criterion. The final sample included 29 teams.

### Team composition

Each multidisciplinary team consisted of five to nine persons and replicated the standard trauma room staffing structure used in each participating hospital, meeting the minimum staffing requirements of the S3-Polytrauma guidelines [3]. Teams consisted exclusively of members from the same hospital, ensuring no cross-institutional mixing of team members.

### Training intervention

All participants completed a standardized, competency-based 6-hour trauma room team training conducted in the actual trauma rooms of the participating trauma centers. Each session used the existing clinical infrastructure of the respective hospitals, including medical equipment and facilities familiar to the participants.

Training was led by an experienced instructor with extensive expertise in trauma care, ATLS instruction, Crew Resource Management (CRM), and medical education, supported by at least two additional co-trainers to ensure standardized scenario flow, simulator operation, and real-time management of vital signs and technical parameters.

The training consisted of five trauma scenarios replicating real-life trauma room conditions, based on anonymized trauma cases from a university hospital. Scenarios covered the full trauma care pathway from patient arrival to clinical handover and included authentic vital signs, diagnostic processes and results (ultrasound, X-ray, CT, blood gas analysis), and therapeutic interventions. A high-fidelity human patient simulator (Smart STAT Professional Simulator, Simulaids Inc., USA; distributed by Ambu, Bad Nauheim, Germany) was used. Injury patterns were simulated using modeling wax and makeup. Vital signs and dynamic physiological responses to team interventions were displayed in real time using REALTi 360 software (3B Scientific GmbH, Germany; formerly iSimulate Pty Ltd, Australia).

### Training formats and standardization

Both training formats, algorithm-based training (control group) and checklist-based training (intervention group), were grounded in ATLS standards [2] and the German S3 Polytrauma Guidelines [3], and incorporated CRM principles [6]. Core competencies targeted in both formats included effective communication, situational awareness, leadership, task delegation, and teamwork strategies such as closed-loop communication and the “10-for-10” principle.

Both formats followed an identical pedagogical structure consisting of a standardized briefing, hands-on simulation, and a structured debriefing. The duration of training, learning objectives, scenario content, instructor team, and targeted competencies were identical across both groups. This design ensured that any observed differences could be attributed to the presence or absence of the checklist component rather than to differences in training intensity, instructor behavior, or curricular content.

### Checklist component (Intervention group)

The key difference between groups was the integration of the Trauma Room Manual (TRM) with its seven standardized checklists in the checklist-based training group. The checklists covered:

1. telephone admission,
2. team preparation,
3. prehospital-to-hospital handover,
4. primary survey,
5. secondary survey,
6. diagnostic prioritization, and.
7. intensive care unit (ICU) handover [16].

The checklists emphasized high-risk, safety-critical steps prone to omission and operationalized two procedural principles: the do–confirm and read–do principles. The do–confirm principle required team members to complete tasks before confirming them during a structured review, implemented through a challenge–response format in which the trauma leader verified each step. Responses were categorized as green (no issues; proceed) or red (critical problem requiring immediate intervention). The read–do principle applied to selected checklist items requiring immediate action upon being read, ensuring that critical interventions were not delayed [16].

While elements such as team time-outs and task delegation are inherent to ATLS, the checklist-based training placed greater emphasis on explicitly defined communication checkpoints and standardized verbal confirmation of critical actions.

### Measurement

Following Floren et al. (2018), SMMs were defined as the degree of similarity (convergence) in team members’ knowledge and expectations regarding teamwork-related aspects, specifically task responsibilities and communication patterns [7]. In line with SMM theory, SMMs were operationalized as within-team similarity rather than as individual mean levels, reflecting the extent to which team members hold a common understanding of team processes relevant for effective coordination in trauma care.

Participants completed a two-part questionnaire consisting of a demographic survey and an SMM instrument.

### Demographic survey

Twelve items captured key demographic characteristics, including profession, years of professional experience, age group, and gender.

### SMM questionnaire

Teamwork-related SMMs were assessed using a questionnaire adapted from the instrument developed by Nakarada-Kordic et al. (2016) [23], which was originally designed to capture shared understanding of team processes and communication in healthcare teams. For the present study, the instrument was contextually adapted to the trauma room setting with input from subject matter experts in trauma care. Item wording was modified to reflect trauma-specific workflows and interprofessional team structures, while retaining the original conceptual domains and response formats.

The adapted version comprised two subscales:

- Task responsibility: 26 items assessing participants’ understanding of individual roles, task allocation, and responsibility distribution within the trauma team, including 21 single-choice and 5 multiple-choice items.
- Team communication: 28 multiple-choice items assessing expectations regarding communication patterns, information exchange and coordination.

In line with the theoretical conceptualization of SMM, individual questionnaire responses were aggregated at the team level, and similarity indices were calculated to reflect the degree of convergence among team members’ perceptions within each team. Higher similarity scores indicate a higher degree of sharedness in team members’ mental representations. Team-level similarity scores were computed separately for the total scale and for each subscale (task responsibility and team communication) at both measurement points (pre- and post-training).

Data were collected digitally using tablet devices (iPads, Apple Inc.), with paper-based backup forms that were digitized post-training.

### Similarity scores

This study included three dependent variables serving as proxies for the degree of SMM within trauma room teams, each assessed at two time points (pre- and post-training):

1. Task Responsibility Similarity Score, reflecting the alignment in team members’ understanding of individual roles and responsibilities.
2. Communication Similarity Score, assessing the alignment in communication patterns among team members, specifically regarding who is expected to communicate completed tasks or clinical problems to whom.
3. Total Mental Model Similarity Score, an aggregated indicator of the team’s overall SMM, capturing collective cognitive alignment across task responsibility and communication domains.

Similarity scores reflected the degree of response alignment across team members and were operationalized as proportions of agreement derived from binary item-level coding. For single-choice items (predominantly task responsibility), agreement between two team members was coded as 1 (e.g., assigning the same individual as responsible for a task) and disagreement as 0. The similarity score for each question was computed by summing agreement scores and dividing by the maximum possible number of agreements, following the approach of Nakarada-Kordic et al. (2016) [23].

For multiple-choice items (predominantly communication patterns), similarity scores were calculated based on the proportion of overlapping responses among team members, as outlined by Sætrevik and Eid (2014) [24]. These differing scoring formats reflect the conceptual and methodological distinctions between the two domains and influence both baseline similarity values and potential for change.

Similarity scores for individual items were first aggregated at the domain level to produce domain-specific similarity scores for each team at each measurement time point. These domain-specific scores were then aggregated to calculate an overall similarity score for each team. The resulting team-level similarity scores ranged from 0 to 1 (equivalent to 0–100% agreement), with higher values indicating greater sharedness, and served as dependent variables in the repeated-measures ANOVA to evaluate the effects of checklist-based and algorithm-based training on the enhancement of SMM over time (Supplement 1).

### Statistical methods

Data were exported from SurveyMonkey (SurveyMonkey Inc., USA) and processed using Microsoft Excel (version 16.78) prior to statistical analysis. Statistical analyses were conducted using IBM SPSS Statistics (Version 29.0.1.0; IBM Corp., Armonk, NY, USA). Descriptive statistics were calculated for demographic and outcome variables. Baseline demographic characteristics were compared at the individual level using chi-square tests for categorical variables and independent-samples t-tests for continuous variables to assess potential baseline imbalances between groups.

Teams were randomized to training conditions; although questionnaire data were collected at the individual level, responses were aggregated to team-level similarity scores, which constituted the unit of analysis. Team-level similarity scores served as dependent variables. Training effects were analyzed using repeated-measures analysis of variance (RM-ANOVA) with time (pre-training vs. post-training) as the within-subjects factor and training group (checklist-based vs. algorithm-based) as the between-subjects factor. Separate RM-ANOVAs were conducted for the Task Responsibility Similarity Score, the Communication Similarity Score, and the Total Mental Model Similarity Score. The time × group interaction was used to examine whether changes in SMM similarity differed between training formats.

Model assumptions [25] were inspected visually using Q–Q plots and residual diagnostics and were deemed acceptable for exploratory inference. As similarity scores are bounded proportions (0–1) and the sample size was limited, analyses are interpreted as exploratory. As the within-subject factor (time) had only two levels, the assumption of sphericity was not applicable. Statistical significance was set at α = 0.05 (two-tailed). Effect sizes were reported as partial eta squared (η²p) and interpreted according to Cohen’s guidelines (0.01 = small, 0.06 = medium, 0.14 = large) [26, 27].

## Results

**Fig. 2:**
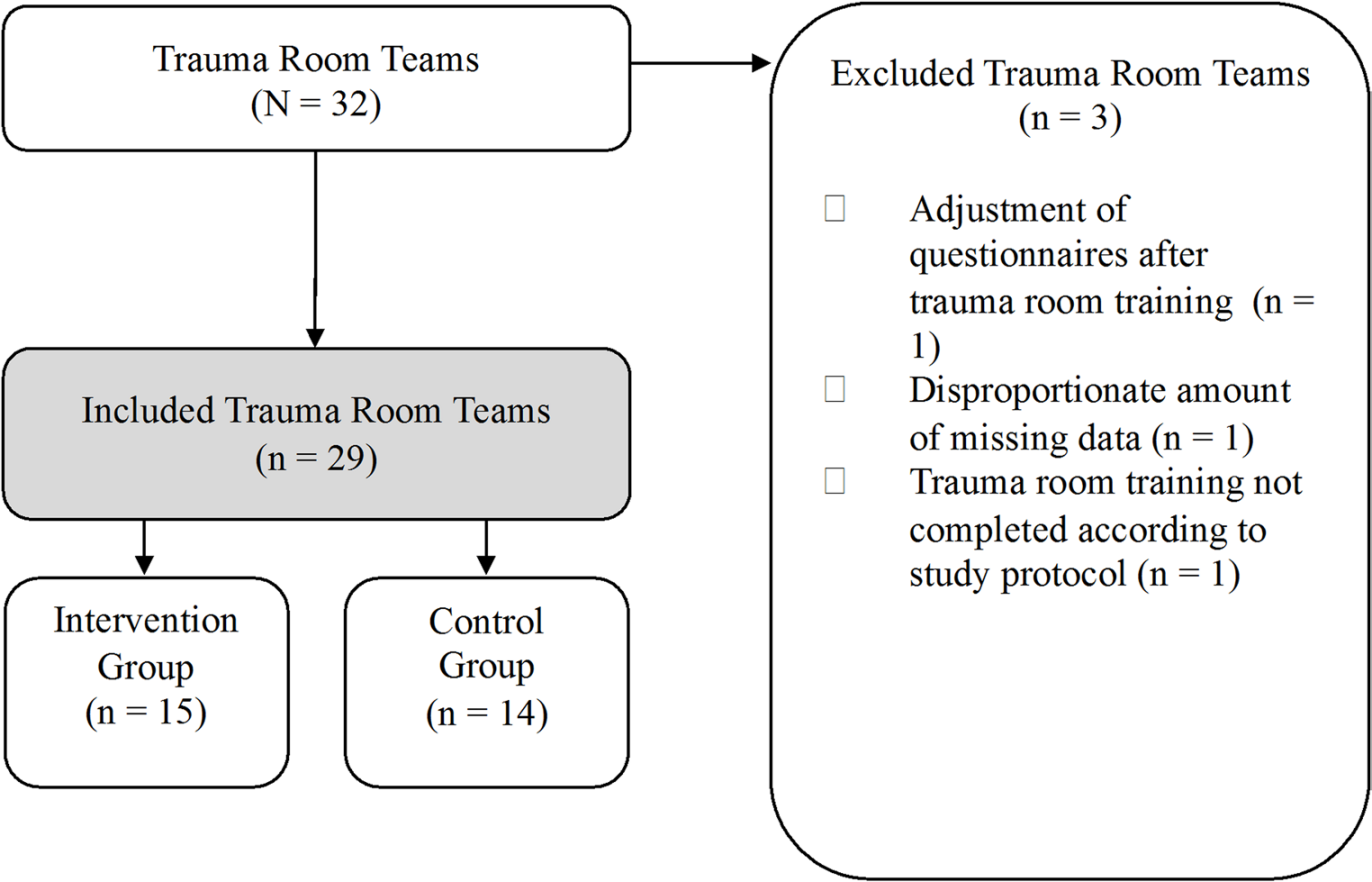
Flowchart

Thirty-two trauma room teams initially participated in this study **(**Fig. 2**).** Three teams were excluded due to early piloting, protocol deviations, or incomplete data, resulting in a final sample of 29 teams comprising 186 participants. Baseline demographic characteristics did not differ between the two groups (Table 1, all *p* > .05).

**Table 1:** Baseline characteristics of study participants

Variable	Intervention group (*n* = 95)	Control group (*n* = 91)	*p*
**Gender**			0.70
Female	60 (63.2)	55 (60.4)	
Male	35 (36.8)	36 (39.6)	
**Age**			0.23
< 30 years	43 (45.3)	30 (33.0)	
30–40 years	30 (31.6)	36 (39.6)	
> 40 years	22 (23.1)	25 (27.4)	
**Occupation**			0.86
Medical doctor	52 (54.7)	51 (56.0)	
Medical staff	43 (45.3)	40 (44.0)	
**Years of professional experience**, M (SD)	7.6 (9.8)	8.6 (8.98)	0.24

Note. Values are reported as number (percentage) unless otherwise indicated. p values were obtained using chi-square tests for categorical variables and independent-samples t tests for continuous variables. Tests were conducted at the individual level

**Table 2:** Descriptive statistics of team-level shared mental model similarity scores (%)

Outcome domain	Training group	*n*	Pre-training, M (SD)	Post-training, M (SD)
Task responsibility	Checklist	15	61.27 (7.48)	73.71 (6.55)
	Algorithm	14	62.26 (7.91)	71.44 (6.06)
Communication	Checklist	15	80.78 (4.16)	82.58 (3.19)
	Algorithm	14	80.42 (3.26)	82.38 (3.21)
Total similarity score	Checklist	15	71.38 (4.68)	78.31 (3.94)
	Algorithm	14	71.67 (3.78)	77.11 (3.35)

Note. Values represent team-level similarity scores expressed as percentages. Inferential statistics are reported in Table 3

**Table 3:** Repeated-measures ANOVA results for team-level shared mental model similarity scores

Outcome domain	Effect	df	F	*p*	Partial η²
Task responsibility	Training group	1, 27	0.07	0.80	0.00
	Time	1, 27	166.18	< 0.001	0.86
	Time × Training group	1, 27	3.79	0.06	0.12
Communication	Training group	1, 27	0.06	0.81	0.00
	Time	1, 27	11.25	0.002	0.29
	Time × Training group	1, 27	0.02	0.89	0.00
Total similarity score	Training group	1, 27	0.11	0.75	0.00
	Time	1, 27	156.50	< 0.001	0.85
	Time × Training group	1, 27	1.27	0.14	0.08

Note. df = degrees of freedom; partial η² = partial eta squared. Training group refers to checklist-based versus algorithm-based training. Time refers to pre-training and post-training measurement

Repeated-measures ANOVA demonstrated significant main effects of time for task responsibility, communication, and total similarity (all *p* ≤.002) (Table 2). No time × training group interactions reached statistical significance; task responsibility showed a trend toward a larger increase in the checklist group (F(1,27) = 3.79, *p*=.06, η²*p* = .12), whereas communication and total similarity did not (Table 3).

## Discussion

### Comparable effects of checklist- and algorithm-based trainings on shared mental models

This cluster-randomized study examined whether checklist-based or algorithm-based in situ simulation training yields differential improvements in teamwork-related SMM in interprofessional trauma teams. Both training formats were associated with significant pre–post gains in total SMM similarity as well as in the subdomains of task responsibility and communication. No significant between-group differences were observed. For task responsibility, the time × group interaction approached statistical significance (*p* =.06; η²*p* = .12), suggesting a potential trend toward larger gains in the checklist group that warrants confirmation in adequately powered studies.

Overall, these findings indicate that structured, competency-based in situ simulation training itself, rather than the specific instructional format, was associated with short-term improvements in team-level cognitive alignment. This interpretation is consistent with prior work demonstrating that simulation-based team training can improve shared understanding and coordination in high-risk clinical and emergency settings, although instruments and analytic approaches vary substantially across studies [17–19, 28–32]. The present study extends this literature by directly comparing two instructional formats within an otherwise standardized training framework and by focusing specifically on teamwork-related SMMs rather than technical task performance.

### Domain-specific patterns: task responsibility vs. communication

Across both training formats, improvements were more pronounced for task responsibility than for communication. Baseline similarity scores were lower for task responsibility and higher for communication, a pattern that must be interpreted cautiously. Methodologically, task responsibility was assessed using single-response items requiring assignment of one responsible team member per task, which constrains agreement and typically results in lower baseline similarity [23]. In contrast, communication items permitted multiple responses, producing higher baseline similarity and reduced ceiling-adjusted change potential [24]. These scoring characteristics partly explain the differing magnitudes of change.

Conceptually, the domains also differ. Task responsibility in trauma teams is strongly protocol-driven and role-defined, reflecting structured workflows anchored in ATLS and national guidelines. Communication practices, by contrast, are more dynamic and influenced by institutional culture, team composition, and situational variability. These structural differences likely affect both baseline convergence and responsiveness to training and should be considered when interpreting domain-specific effects.

### Mechanisms of SMM improvement in trauma teams

Improvements in SMM can be interpreted through collaborative learning and cognitive load frameworks. In high-acuity environments such as trauma resuscitation, teams operate under substantial intrinsic cognitive load. Structured simulation training may reduce extraneous load by clarifying task sequencing, reinforcing role expectations, and promoting standardized communication strategies [33–38]. Repeated, scenario-based practice allows team members to align expectations regarding leadership, information flow, and escalation pathways. In this sense, SMM function as a collective cognitive resource that distributes cognitive demands across the team, supporting anticipatory coordination and reducing the need for corrective communication during time-critical phases of care. Importantly, the observed improvements likely reflect enhanced cognitive alignment rather than format-specific mechanisms.

### Differentiated mechanisms of the training formats

Both training approaches were grounded in ATLS principles and incorporated structured role allocation and CRM-derived communication strategies. These shared components likely explain the comparable improvements observed across groups. Because ATLS already provides a standardized framework for task prioritization and role clarity, structured in situ simulation may have been sufficient to enhance teamwork-related SMMs independent of instructional format. Furthermore, as CRM-related elements are integral to ATLS training, both courses could build on preexisting knowledge structures, an approach associated with effective learning integration [39].

The principal structural distinction between the interventions was the integration of the Trauma Room Manual (TRM) in the checklist-based group. The TRM formalizes verification processes, role confirmation, and structured briefings via explicit checklist use. Theoretically, such cognitive aids may reduce cognitive load by externalizing task sequencing and reinforcing shared representations of responsibilities and priorities.

Consistent with the overall pattern of non-significant interaction effects (with task responsibility approaching significance), the additional checklist structure did not demonstrate a measurable advantage in teamwork-related SMM alignment within the timeframe of this study. Variable checklist adherence during simulation may have attenuated potential format-specific effects, consistent with previously described training–practice gaps [20]. Accordingly, the incremental contribution of formalized checklist integration remains to be clarified in future studies incorporating standardized fidelity monitoring.

### Methodological considerations in the existing literature

Direct comparison of SMM levels and training effects across studies remains limited by substantial methodological heterogeneity. Prior investigations have used qualitative interviews [19], perceptual self-ratings [17], and differing similarity metrics [23, 24], often without standardized pre–post designs or team-level aggregation. Consequently, reported similarity values and effect sizes are not directly comparable. Greater methodological standardization in SMM assessment, particularly in trauma contexts, would strengthen cumulative evidence and allow more precise evaluation of training strategies.

### Clinical and trauma-specific implications

Although SMM similarity represents a cognitive surrogate outcome, trauma resuscitation research has demonstrated that process metrics such as time to completion of the primary survey, time to computed tomography or operative decision-making, and rates of task omission are used as indicators of trauma team performance and resuscitation efficiency [9, 10, 15, 40]. Future studies should therefore examine whether increases in SMM alignment are associated with improvements in such trauma-relevant process measures. Linking SMM similarity to objective performance markers and error patterns (e.g., delayed escalation, missed injuries, communication breakdown at handover) would strengthen clinical interpretability and clarify whether enhanced cognitive alignment translates into measurable improvements in trauma workflow reliability.

### Limitations

Several limitations should be considered when interpreting these findings. First, the study focused exclusively on teamwork-related SMM components, namely task responsibility and communication. The taskwork domain, which is highly standardized through ATLS-based protocols, was not assessed and may respond differently to training interventions. Consequently, conclusions are restricted to teamwork-related cognitive alignment rather than procedural execution. Second, although an a priori sample size calculation targeted medium effects, the final sample (29 teams) may have limited power to detect small between-group or interaction effects. In particular, the study may not have been sufficiently powered to identify subtle differences between checklist-based and algorithm-based training formats. Third, team allocation occurred at the team level, and outcomes were analyzed at the team level. However, hospital-level clustering effects were not modeled due to the limited number of teams per center. Potential residual correlation at the institutional level, therefore, cannot be excluded. Fourth, SMM were assessed using a self-administered questionnaire capturing predefined aspects of team cognition. While this approach allows standardized quantification of similarity, it primarily reflects static representations of roles and communication expectations and may not fully capture dynamic adaptation during unexpected clinical events or role switching in real-time trauma care [21]. Fifth, variation in team size may have influenced similarity scores, as larger teams inherently generate more pairwise comparisons and potentially greater cognitive heterogeneity. Moreover, checklist adherence was not formally measured; trainer observations suggested variability in checklist use across scenarios and teams. Without fidelity monitoring, potential format-specific effects may have been attenuated. Finally, the study operationalized SMM solely in terms of similarity (convergence) without assessing accuracy. High similarity does not necessarily indicate correct or optimal task representations, as highlighted in theoretical models of SMM (e.g., Floren et al. [7]). Moreover, SMM alignment represents a cognitive surrogate outcome. The present study did not assess behavioral performance, process indicators, or patient outcomes; therefore, no conclusions can be drawn regarding downstream clinical effects.

## Conclusion

This cluster-randomized study demonstrates that both checklist-based and algorithm-based in situ simulation training significantly improve teamwork-related SMM in interprofessional trauma teams. Improvements were observed in task responsibility, communication, and overall SMM alignment, with comparable magnitudes across both training approaches. No evidence of superiority of one instructional format over the other was identified. These findings indicate that structured, competency-based simulation training itself is effective in enhancing team-level cognitive alignment in trauma resuscitation settings, independent of the specific instructional format employed. SMM alignment represents a surrogate outcome reflecting team cognition rather than direct clinical performance or patient outcomes. Further research is needed to determine the durability of these effects and to examine whether improvements in SMM are associated with measurable changes in team behavior or clinical processes.

## Data Availability

The data are available from the authors upon reasonable request.

## Abbreviations

ATLS: Advanced Trauma Life Support
ABCDE: Airway, Breathing, Circulation, Disability, Exposure
ANOVA: Analysis of variance
CIRS: Critical Incidence Reporting System
CRM: Crew resource management
ICU: Intensive Care Unit
SOPs: Standard Operating Procedures
TRM: Trauma Room Manual
